# Self-assembly of amino acids toward functional biomaterials

**DOI:** 10.3762/bjnano.12.85

**Published:** 2021-10-12

**Authors:** Huan Ren, Lifang Wu, Lina Tan, Yanni Bao, Yuchen Ma, Yong Jin, Qianli Zou

**Affiliations:** 1 School of Pharmacy, Anhui Medical University, Hefei 230032, China

**Keywords:** amino acids, functional biomaterials, intermolecular interactions, self-assembly

## Abstract

Biomolecules, such as proteins and peptides, can be self-assembled. They are widely distributed, easy to obtain, and biocompatible. However, the self-assembly of proteins and peptides has disadvantages, such as difficulty in obtaining high quantities of materials, high cost, polydispersity, and purification limitations. The difficulties in using proteins and peptides as functional materials make it more complicate to arrange assembled nanostructures at both microscopic and macroscopic scales. Amino acids, as the smallest constituent of proteins and the smallest constituent in the bottom-up approach, are the smallest building blocks that can be self-assembled. The self-assembly of single amino acids has the advantages of low synthesis cost, simple modeling, excellent biocompatibility and biodegradability in vivo. In addition, amino acids can be assembled with other components to meet multiple scientific needs. However, using these simple building blocks to design attractive materials remains a challenge due to the simplicity of the amino acids. Most of the review articles about self-assembly focus on large molecules, such as peptides and proteins. The preparation of complicated materials by self-assembly of amino acids has not yet been evaluated. Therefore, it is of great significance to systematically summarize the literature of amino acid self-assembly. This article reviews the recent advances in amino acid self-assembly regarding amino acid self-assembly, functional amino acid self-assembly, amino acid coordination self-assembly, and amino acid regulatory functional molecule self-assembly.

## Review

### Introduction

Biomaterials play a crucial role in the treatment of diseases and health care and have been widely used in prostheses and drug delivery devices [1]. Clinical applications of biomaterials include the use of metals, ceramics, and polymers to enhance, repair, or replace diseased, damaged, or defective tissue [2]. A few examples are tooth repair, peripheral nerve regeneration, nerve tissue engineering, bone and joint replacement and repair, and regeneration of bone defects, biological scaffolds, and wound healing [3–8]. Although traditional materials provide good structural analogues for native bone and surrounding tissue, they are difficult to mimic the dynamics and complexity of the natural environment [9]. Therefore, it is necessary to develop a new generation of biomaterials to improve strategies for natural tissue structure and functional reorganization.

Peptides and proteins are attractive candidates for functional biomedical materials [10] due to their extensive existence in nature, easy access, and good biocompatibility [11]. Self-assembly refers to the selective and spontaneous formation of one or more well-ordered structures from a complex mixture via noncovalent interactions, including van der Waals forces, electrostatic forces, hydrogen bonds, and stacking interactions [12–13]. Importantly, biomolecules, such as proteins, peptides, or biologically derived molecules, including de novo designed peptides or nucleotides, can be self-assembled [14]. Proteins are direct functional performers of countless interactions between living organisms and the outside world, and are susceptible to changes in pH value, ionic strength, and temperature [15]. Peptides have similar biocompatibility and diversity as proteins, but have better availability, can be obtained in a larger scale, and have higher stability and durability than proteins. Furthermore, some peptides may have the same function as proteins by retaining functional sequences [16]. Many studies have reported that proteins and peptides can be assembled into various nanostructures, such as nanowires [17], nanofibers [18], nanospheres [19], nanovesicles [20], nanogels [21], nanobelts [22], and nanotubes [23]. Self-assembly not only conveys higher stability and mechanical strength to proteins and peptides, but also further enhances their natural activity and function due to the collective behavior of aggregates [24]. However, protein and peptide assemblies exhibit disadvantages, such as difficulty in obtaining high quantities of materials, corresponding high costs, and in some cases polydispersity and purification limitations. When using proteins and peptides as functional materials it can be difficult to arrange assembled nanostructures at both microscopic and macroscopic scales [10]. Also, fine manipulation of noncovalent interactions and corresponding peptide and protein nanostructures remains a huge challenge [24].

Amino acids are the major components of all naturally occurring peptides and proteins [25]. Amino-acid-based nanostructures are self-assembled from the simplest building blocks in the biological system environment and are the smallest component of the bottom-up approach [26]. Amino acids and their derivatives can be self-assembled into ordered nanostructures through noncovalent interactions, including electrostatic, π–π stacking, van der Waals, and hydrophobic interactions. The self-assembly of single amino acids has the advantages of low synthesis cost, relatively easy modeling [27], and excellent biocompatibility and biodegradability in vivo [28] compared with the self-assembly of large molecules, such as proteins and peptides. Importantly, amino acids or amino acid derivatives may be self-assembled with other components to form functional architectures, such as drug delivery systems, light collection systems, and imaging systems. However, using these simple building blocks to design attractive materials remains a challenge due to the simplicity of the amino acids [26]. Most articles about self-assembly focus on peptide and protein self-assembly [15,29]. Therefore, it is of great significance to systematically summarize the latest advances in the field of amino acid self-assembly.

**Figure 1:**
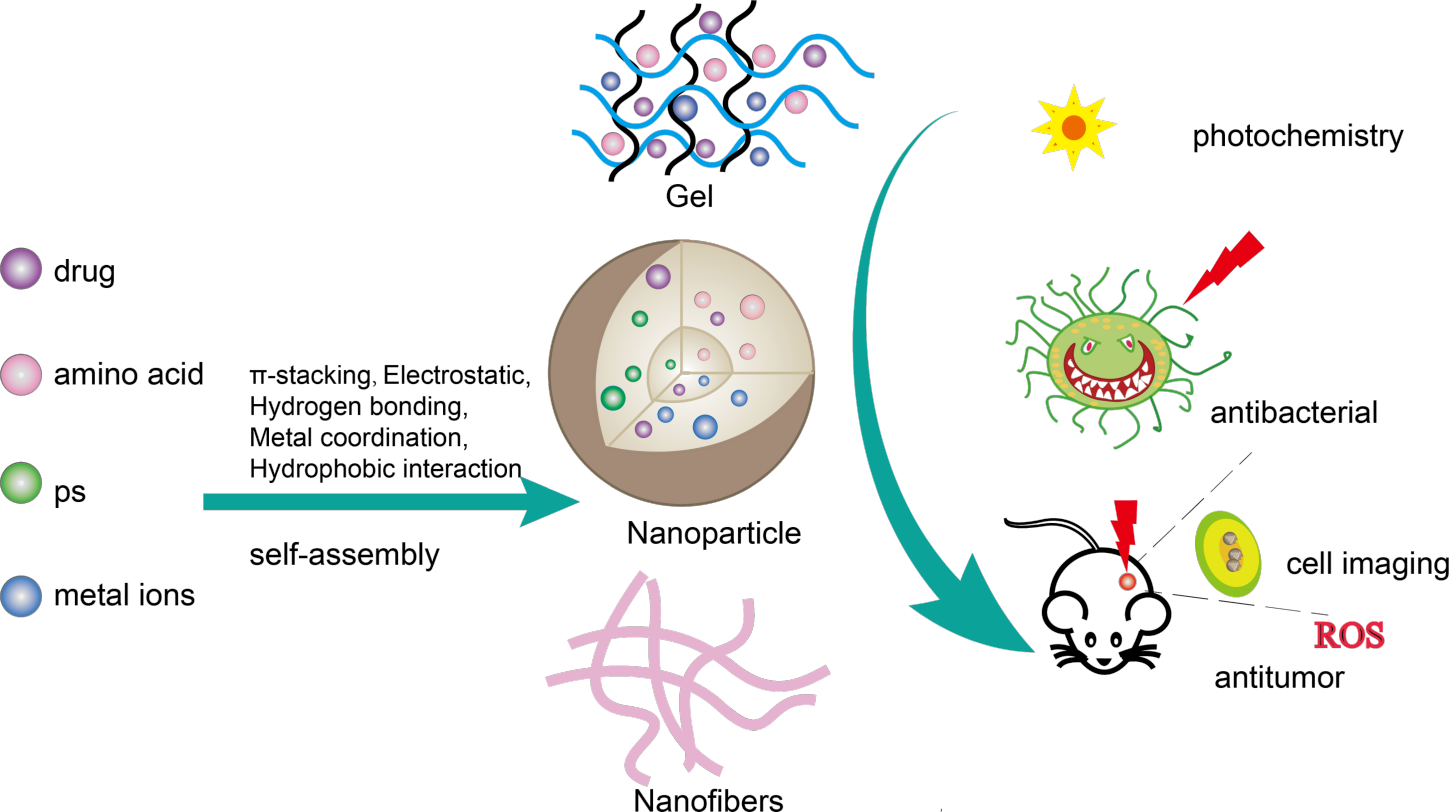
Schematic diagram of amino acid regulatory self-assembly (amino acid–drugs, amino acid–photosensitizers, amino acid–metal ions, multi-component collaborative self-assembly) as a general strategy for functional biomaterials research.

In this review, we highlight the latest advances in amino acid self-assembly. These self-assembly methods mainly focus on single amino acid self-assembly, modified amino acid self-assembly, amino acid and metal ion coordination self-assembly, and amino acid and functional molecule self-assembly (drug, photosensitizer) (Figure 1). In this paper, the self-assembly of single amino acids is discussed first. We then discuss the co-assembly of amino acids and their derivatives with functional components including metal ions, photosensitizers (PS), and pharmaceuticals. Finally, the applications of these assemblies in various systems are introduced.

### Self-assembly of amino acids

Self-assembly is the process of creating high-level functional structures from simple building blocks, such as amino acids, peptides, proteins, and phospholipids [30]. Noncovalent bonds and molecular forces play a key role in self-assembly, including hydrogen bonds, hydrophobic bonds, van der Waals force, ionic bonds, π–π stacking, and electrostatic forces [31]. Importantly, amino acids are simple building blocks that provide relevant noncovalent interactions to construct complex supramolecular assemblies [32–33]. Twenty natural amino acids are used by cells to synthesize peptides and proteins.

A single amino acid can be self-assembled. For example, Adler-Abramovich et al. [34] showed for the first time that phenylalanine, a single aromatic amino acid, can form ordered fibrillar assemblies at the nanoscale. This component exhibits regular aggregate properties through hydrogen bonding and ion interaction, which are highly similar to those of amyloid components, suggesting that it may be associated with the etiology of amyloid-related diseases. Besides, the resulting structure is as toxic to cells as other amyloid structures. Their subsequent study continued to demonstrate that other single amino acids and metabolites, including cystine, tyrosine, and adenine, also self-assemble to form elongated and fibrillar structures at the nanoscale [35]. Likewise, the characteristics of these combinations suggest that all assembled ultrastructures formed from various metabolites exhibit amyloidosis. These metabolites not only self-assemble into supramolecular amyloid fiber structures, but also have significant apoptotic effects on neuron model cells. Singh et al. [36] showed that the hydrophobic interaction between phenylalanine (Phe) rings may play a major role in the self-assembly process. Interestingly, their study also revealed that ᴅ-Phe changes ʟ-Phe fibrous state to flakes, which do not propagate further and do not seed ʟ-Phe, suggesting that ᴅ-Phe may be a potential therapeutic molecule for phenylketonuria. Bera et al. [37] studied the effect of chiral on aromatic amino acid self-assembly. They found that the hybrid ᴅʟ system (racemate) alters the morphology and dynamics of the assemblies. For example, either ʟ-phenylalanine or ᴅ-phenylalanine can form amyloid fibers, but the ᴅʟ system shows a crystalline sheet-like assembly. Interestingly, their group also identified the optical properties of single amino acids during assembly, demonstrating the intrinsic fluorescence of amyloid structures of metabolites such as adenine, tryptophan, tyrosine, and phenylalanine which can be used to detect living cells [38]. Gour et al. explored the self-assembly of nonaromatic amino acids. They first reported the ability of nonaromatic single amino acids, cysteine and methionine, to spontaneously self-assemble to form protein-like aggregates, which are very long and fibrous and may be cytotoxic to human neuroblastoma cells [39]. These studies provide a new paradigm for metabolic disorders caused by single amino acids in amyloid-associated diseases.

**Self-assembly of functionalized amino acids.** There are only 20 natural amino acids, so it is very limited to rely on the self-assembly of natural amino acids. Self-assembly with modified amino acids is of great interest, most of which focuses on 9-fluorenylmethoxycarbonyl (Fmoc)-terminated materials [40–43]. Due to the inherent hydrophobicity and aromaticity of Fmoc, many Fmoc-modified amino acids exhibit relatively fast self-assembly kinetics, excellent physical and chemical properties, and great potential in cell culture, photocatalysis, drug delivery, and antibacterial applications [27].

The Fmoc modification of a single amino acid is the simplest building block, among which Fmoc-phenylalanine is the most studied due to its good hydrocoagulant properties [27] and good antibacterial activity [44]. Hydrogels of other amino acids modified by Fmoc, such as Fmoc-tryptophan, Fmoc-methionine, and Fmoc-tyrosine have also been shown to have antimicrobial activity and to be selectively resistant to Gram-positive bacteria [45].

The combined self-assembly strategy can provide different functions for amino acid assembly. Fmoc-phenylalanine and Fmoc-leucine were co-assembled, with Fmoc-phenylalanine as the hydrocoagulant and Fmoc-leucine as the antimicrobial unit. The resulting hydrogel selectively killed *Staphylococcus aureus* by breaking the cell wall and membrane and had good biocompatibility. After 20 h of incubation, approximately 95% of S*taphylococcus aureus* bacterial proliferation was effectively inhibited [46]. A novel supramolecular self-assembled hydrogel was prepared by mixing Fmoc-ʟ-phenylalanine (Fmoc-ʟ-Phe) with oligo(thiophene ethynylene)-ᴅ-phenylalanine (OTE-ᴅ-Phe), an amino-acid-modified conjugated oligomer [47]. Fmoc-ʟ-Phe/OTE-ᴅ-Phe formed yellow and transparent hydrogels through hydrogen bonding, van der Waals interactions, π–π interactions, and hydrophobic interactions, showing thicker and rougher nanofibers, which had obvious advantages in effectively capturing model bacteria methicillin-resistant *S. aureus* and *Escherichia coli*. When Fmoc-ʟ-Phe/OTE-ᴅ-Phe is coated on the surface, it also exhibits a strong ability to specifically kill methicillin-resistant *S. aureus*. In addition, Chakraborty et al. [43] designed a gelling agent containing two Fmoc groups, Fmoc-lysine and Fmoc-aspartic acid, by two-step self-assembly, which had an ultra-low critical gelling concentration and good mechanical properties and can be used for 2D/3D cell scaffolds and the production of conductive soft composites. In addition, Fmoc-amino acids can be co-assembled with drugs to play different therapeutic effects as drug delivery carriers. The encapsulation of the antibiotic aztreonam (ATZ) in the Fmoc-phenylalanine (Fmoc-F) hydrogel expands the antibacterial range of Fmoc-F, which can continuously release ATZ and Fmoc-F in the wound [48]. The AZT encapsulated Fmoc-F hydrogel was used in established wound infections, which slowly degraded within 24 h and were topically coated with AZT Fmoc-F hydrogel two days after infection, resulting in a 1000-fold reduction in bacterial load compared to untreated wounds. Aztreonam is specific against Gram-negative bacteria and aztreonam-encapsulated Fomc-F hydrogels antagonize *Pseudomonas aeruginosa* and enhance Fomc-F antimicrobial activity. Salicylic acid is loaded in Fmoc-ʟ-phenylalanine hydrogel, which can play a role against Gram-positive bacteria, and the drug release behavior changes at different temperature and pH values [49]. Rizzo et al. studied camptothecin loaded into Fmoc-F hydrogels in the presence of haloalkyl nanotubes. Importantly, this hybrid co-assembly method could not only control the release of camptothecin in the form of active lactone, but also showed significant inhibitory activity on cancer cells [50].

Furthermore, naphthalimide (NI) derivatives can also be used as end-capping materials for amino acids. Naphthalimide has unique photophysical properties and photostability as a luminescent material for aggregation-induced emission (AIE), which can display high emission properties in the aggregated state and can be used for imaging [51]. Importantly, NI exhibits hydrophobicity and π–π stacking due to the aromatic moieties, and is prone to dynamic aggregation, which can be used in self-assembled construction units [52]. For example, Hsu et al. [53] self-assembled NI and phenylalanine to produce hydrogels driven by hydrogen bonds and π–π interactions and to form microfiber three-dimensional networks at 1 wt % and pH 7.4. The microfibers have AIE properties and strong blue emission under an ultraviolet lamp. Ni-terminated hydrogels of NI-Phe exhibit viscoelasticity with a storage modulus (*G*') value of about 2000 Pa, and can be used for imaging three-dimensional cytoskeletal materials. After human mesenchymal stem cells (hMSCs) were cultured for 72 h in the 3D fiber hydrogel, the cell viability in the 3D gel was subsequently verified. Only a few dead cells were observed, indicating that hMSCs had a high viability in the supramolecular hydrogel formed by NI-Phe. Sarkar et al. synthesized amphiphilic molecules containing NI and histidine which formed fluorescent organic nanoparticles with J-aggregation in water/DMSO, exhibiting high emissivity upon aggregation. These particles were used for the selective sensing of Fe^3+^ within cancer cells and imaging of Fe^3+^ [54].

**Amino-acid-coordinated self-assembly.** Coordination-driven self-assembly is a supramolecular self-assembly method based on metal-coordination bond formation, which has the advantages of fewer steps, fast final product, easy assembly, self-calibration, and no defects [55]. The prepared components may further be used as modular “building blocks” for building higher-order upper structures with increased complexity and functionality [56]. Metal coordination can become a strong interaction due to its near-covalent characteristics compared to the common noncovalent interactions in self-assembly, such as hydrophobic interactions, van der Waals force, hydrogen bonds, ion attraction, and π–π stacking [57].

Cystine (Cys) can provide carboxyl and amino groups with which it can coordinate with equimolar amounts of cadmium ions (Cd^2+^) to form a three-dimensional crystal of Cys/Cd nanorods. Then, upon the introduction of Na_2_S, the Cys/Cd template mediates the mineralization of cadmium sulfide (CdS) into a layered CdS quantum dot structure, finally making a simple bionic daylight antenna with sustainable photocatalytic performance [58]. In addition, Liu et al. [59], inspired by the cystine pathological biomineralization process, developed a zinc-directed cystine assembly to mimic chloroplast photosynthesis. Zn^2+^ promotes rapid nucleation of cystine crystals and regulates crystal morphology through splitting growth mechanisms. Scanning electron microscopy and transmission electron microscopy images show that the assembly is layered and has a three-dimensional spherical structure with porous and stacked nanorods (width 50 nm, length 200 nm). During the self-assembly process, tetrakis(4-sulfonatophenyl)porphine and alcohol dehydrogenase may be incorporated into Cys microspheres, resulting in hybrid microspheres with photocatalytic and biocatalytic activities. In addition, Cys/Zn microspheres were modified with CO_3_^2−^-doped ZnS nanocrystals by a hydrothermal treatment, and then glutamic acid dehydrogenase was encapsulated in the Cys/Zn framework as a guest molecule to obtain the original pigment model, which can achieve methyl violet (MV^2+^) photoreduction, CO_2_ photoreduction, NADH formation, and hydrogen release [60]. In addition, histidine has site-specific metal ion coordination with an imidazole ring [61]. Han et al. [62] self-assembled metal nanoparticles with good catalytic activity by coordination of histidine derivatives *N*-(benzyloxycarbonyl)-ʟ-histidinohydrazide with zinc ions on a minimum design principle. The catalytic performance of *p*-nitrophenyl acetate hydrolysis to *p*-nitrophenol was evaluated by monitoring the absorbance of *p*-nitrophenol at 400 nm. The catalytic formation of *p*-nitrophenol started within the first three minutes and then gradually increased. The catalytic rate constant of metal nanoparticles was higher than that of lipase (6.00 × 10^−2^ and 6.95 × 10^−3^ s^−1^, respectively). In addition, the substrate affinity of metal nanoparticles (1.53 mM) is comparable to that of natural lipase (1.27 mM).

Metal ions, especially silver ions (Ag^+^), have been widely studied regarding antibacterial, antifungal, antiviral, anti-inflammatory, anti-angiogenic, and antitumor activities [63–64]. Silver nanoparticles (Ag NPs) hold great promise due to their broad-spectrum and robust antimicrobial properties [65]. The main mechanism is that Ag nanoparticles diffuse into cells and destroy cell walls [66]. However, Ag NPs are cytotoxic, which limits their application [67]. Song et al. [68] developed a broad-spectrum antimicrobial metallohydrogel based on Ag^+^-coordinated Fmoc-amino acid self-assembly and local mineralization. The antibacterial activity of the amino acid metallohydrogel against *Escherichia coli and Staphylococcus aureus* was better than that of Ag^+^ solution. In addition, Fmoc-amino acid metallohydrogels showed fewer toxicological side effects and were highly biocompatible than Ag^+^ in the administered dosage range. Furthermore, Fmoc-amino acid metallohydrogels tested under the same conditions showed significantly better wound healing than silver sulfadiazine cream and the control group.

It has been shown that a novel protein-based nanoparticle with enhanced photothermal effect has been obtained for antitumor therapy using metal ions, proteins, and photosensitizers as building blocks [69]. The integration of metal ions significantly improved the structural stability and photothermal properties of the nanoparticles [69]. The use of amino acids coordinated with metal ions and the encapsulation of guest molecule photosensitizers have also achieved encouraging results. Zhang et al. [70] developed an antitumor photodynamic therapy (PDT) nanoparticle based on the coordination of modified amino acids and metal ions. The amphiphilic amino acid 9-fluorenylmethoxycarbonyl-ʟ-leucine (Fmoc-ʟ-L) and Mn^2+^ were coordinated to encapsulate the hydrophobic photosensitive drug chlorin e6 (Ce6) into a supramolecular system to obtain Fmoc-ʟ-L/Mn^2+^/Ce6 nanoparticles (FMC NPs). Because of the strong coordination of Mn^2+^, Fmoc-ʟ-L, and Ce6, a yield of 36 wt % can be obtained. After the uptake of FMC NPs by cancer cells, Mn^2+^ and Ce6 can be released in response to intracellular high levels of glutathione (GSH). Magnetic resonance imaging (MRI) results showed an almost complete elimination of the tumor three days after injection. At the same time, the formation of Mn^2+^ and GSH can decrease the level of intracellular GSH and promote the production of reactive oxygen species. In addition, Mn^2+^ combined with GSH can also be used for MRI diagnosis and treatment. By using these features, FMC NPs showed better antitumor PDT effects. In addition to Mn^2+^, Li et al. [71] self-assembled Fmoc-L, Fmoc-H, and *N*-benzyloxycarbonyl-ʟ-histidine-ʟ-phenylalanine with zinc ions to form Fmoc-H/Zn^2+^ and Z-HF/Zn^2+^ nanoparticles (approx. 70 nm in size). Then, Ce6 was encapsulated and the Ce6 loading of Fmoc-H/Zn^2+^/Ce6 and Z-HF/Zn^2+^/Ce6 was greater than 50.0% in both cases, and the encapsulation efficiency was greater than 99.0%. The Fmoc-H/Zn^2+^/Ce6 and Z-HF/Zn^2+^/Ce6 nanoparticle assembly were based on coordination and other noncovalent interactions which are sensitive to environmental changes. They demonstrated the robustness of metal nanoparticles under physiological conditions and the abrupt responsive release upon pH and glutathione changes. The half-life of Ce6 in Fmoc-H/Zn^2+^/Ce6 (8.71 h) and Z-HF/Zn^2+^/Ce6 (6.33 h) was much longer than that of unencapsulated Ce6 (3.69 h), according to the fitting results of the pharmacokinetic model. To investigate the in vivo distribution of metal nanoparticles in tumor-bearing mice, mice injected with metal nanoparticles or unencapsulated Ce6 had strong fluorescence signals throughout the body 2 h after injection. Fmoc-H/Zn^2+^/Ce6 or Z-HF/Zn^2+^/Ce6 showed strong fluorescence at the tumor site 24 h after injection. In contrast, no significant fluorescence was observed in mice injected with unencapsulated Ce6 12 h after injection. In addition, metal nanoparticles induce an effective tumor ablation, while unencapsulated Ce6 only partially inhibits tumor growth.

Curcumin is a promising natural antitumor drug, which can inhibit the transformation, proliferation, and migration of tumor cells through various ways, and has anti-angiogenic activity and good biocompatibility [72]. However, the poor water solubility and low bioavailability of curcumin hinder its direct application [73]. Therefore, it is necessary to develop an encapsulation system to improve the bioavailability of curcumin in the tumor microenvironment in order to achieve effective delivery of curcumin and improve the therapeutic effect [74]. Li et al. [75] dissolved 9-Fmoc-ʟ-histidine (Fmoc-H) in hexafluoropropofol or dilute hydrochloric acid and self-assembled curcumin nanoparticles, B-Cur NPs (180 ± 25 nm in size) and S-Cur NPs (80 ± 16 nm in size), by coordination with zinc chloride and curcumin, respectively, in combination with a variety of noncovalent interactions. The stability of curcumin increases with the formation of B-Cur NPs or S-Cur NPs. Even after an extended incubation time of 720 h, the curcumin content in B-Cur NPs and S-Cur NPs was approximately 67% and 77%, respectively. Furthermore, the release of curcumin from B-Cur NPs and S-Cur NPs can be effectively triggered by pH and redox stimulation, facilitating tumor therapy. Selective tumor accumulation was still observed up to 12 h in mice injected with fluorescently labeled (FL-labeled) B-Cur NPs or S-Cur NPs, while mice injected with FL-labeled curcumin showed no significant tumor accumulation after 4 h. In addition, antitumor activity experiments showed that the nanoparticles had higher cytotoxicity and better tumor inhibition than curcumin and did not decrease the biocompatibility of the drug. A tumor inhibition rate of 33.2% was observed in mice treated with curcumin (25 mg·kg^−1^). In contrast, the tumor inhibition rate in mice treated with the corresponding concentration of S-Cur NP (25 mg·kg^−1^ of curcumin) reached 69.6%. Therefore, this method overcomes the obstacles of using pure curcumin in clinical applications and provides a new view for curcumin to effectively treat tumors.

**Amino-acid-modulated self-assembly of functional molecule.** Amino acids can be co-assembled with photosensitizers to form a variety of complex system structures, such as light collection systems, bionic systems, and delivery systems for PDT. Photodynamic therapy is a novel, noninvasive antitumor therapy based on photosensitizers, light, and oxygen [76]. However, the inherent disadvantages of PS, such as hydrophobicity and easy aggregation under physiological conditions, reduce its therapeutic efficiency [77]. Using nanotechnology to encapsulate PS in nanoparticles can effectively solve this problem, improve the bioavailability of PS, and achieve targeted delivery of PS to tumor tissues [76]. Liu et al. [78] designed photosensitizer delivery systems by self-assembling cationic diphenylalanine (H-Phe-Phe-NH_2_ HCl, CDP) or 9-fluorenylmethoxycarbonyl-ʟ-lysine (Fmoc-ʟ-Lys) with Ce6 into nanoparticles (CCNPs and FCNPs, respectively). Intermolecular hydrophobic and π–π interactions contribute to co-assembly. FCNPs nanoparticles and free Ce6 were injected into the caudal vein of MCF7 tumor-bearing nude mice at different times. The assembled NPs selectively accumulated in the tumor. In addition, the fluorescence remained at the tumor site for 24 h, indicating a long residence time. However, a weak fluorescence signal was observed in the tumor site of the mice treated with free Ce6. Designing nanoplatforms responsive to pH, enzymes, and photothermal stimuli is critical to enhance cellular uptake and control PS release [79–81]. Nanoplatforms responsive to pH undergo conformational changes through various mechanisms, such as protonation, charge inversion, or chemical bond cleavage, promoting tumor-specific cellular uptake or drug release [82]. Sun et al. [83] coupled tryptophan-glycine (WG) to hydrophobic porphyrins (P) by an amidation reaction to obtain pH-responsive nanoparticles (PWG) capable of spontaneous assembly under physiological conditions. Interestingly, since glycine provides carboxyl groups and is acid-sensitive, when the nanoparticles reach the tumor site, the acidity increases and the protonation of PWG promotes the formation of intermolecular hydrogen bonds and induces the conversion of nanoparticles to nanofibers. In addition, the nanoparticles exhibited significant long-term fluorescence after intravenous injection, maintained for 168 h, and the fluorescence intensity in the tumor remained above 64% after 168 h, indicating that the PWG nanostructures exhibited high accumulation and ultra-long tumor retention effects. Importantly, animal experiments demonstrated complete eradication of tumor in mice after injection of PWG NPs and laser irradiation, demonstrating the efficacy of PWG nanoparticles in vivo.

Indocyanine green (ICG) is widely used in diagnosis and treatment because of its strong absorption ability in the near-infrared region [84]. However, ICG has poor stability and a short half-life, thus limiting its use in photothermal therapy. Liu et al. [85] developed a nanoparticle based on phenylalanine, geniposide, and ICG for antitumor photothermal therapy. Geniposide is a natural crosslinking agent that provides strong covalent interactions to enhance stability. In addition, they attached disulfide groups to phenylalanine in response to glutathione. The obtained nanoparticles (GDSP) have high stability and can improve the photostability and maintain the photothermal conversion efficiency up to 32.0%. After entering the tumor cells, the nanoparticles convert light into heat under a laser irradiation of 808 nm and effectively kill the tumor cells.

Inspired by natural photosynthesis, artificial light systems consisting of photosensitizers and biomolecules, such as proteins, peptides, and DNA have received extensive attention in recent years [86]. Liu et al. [87] used electrostatic force to adsorb tetrakis(4-sulfonatophenyl)porphine (TPPS) molecules on the surface of 9-fluorenylmethoxycarbonyl-ʟ-lysine (Fmoc-ʟ-Lys) self-assembled nanofibers such that the nanofibers were assembled into sea-urchin-like microspheres. Fmoc-ʟ-Lys nanofibers act as templates to regulate the self-assembly of pigments. Sea-urchin-like structures facilitate light collection due to enhanced absorption cross sections and exciton energy transfer. In addition, Liu et al. [88] combined chemical reactions and manufactured bionic photobacteria based on self-assembly of amino acids and porphyrins. First, Fmoc-ʟ-Lys was self-assembled into a nanofiber template, then the ε-amino group on the surface of the Fmoc-ʟ-Lys fiber reacted with 3,4-dihydroxyphenylalanine (DOPA) melanin through a Schiff base reaction to form an adhesive layer, and Fmoc-ʟ-Lys/DOPA fiber simulated an antenna to capture light. As a photosensitizer, Sn(IV)tetrakis(4-pyridyl)porphyrin (SnTPyP) was combined with the photocatalyst Co_3_O_4_ NPs by coordination bonds and electrostatic interaction onto the adhesive fibers. Therefore, a simple and robust bionic cyanobacteria model with excellent catalytic activity and sustainability was obtained. In addition, amino acids were co-assembled with phthalocyanines to improve their functionality. Han et al. [89] used histidine derivatives, 9-fluorenylmethoxycarbonyl-ʟ-histidine, (Fmoc-His-OH) co-assembled with phthalocyanine tetrasulfonic acid to form nanocapsules to mimic the function of photooxidase. The synergistic effects of various molecular interactions are the cause of the formation of nanocapsules, which can precisely regulate the assembly of photosensitizers and limit their severe self-aggregation. Dopamine was chosen as the model substrate to illustrate the photooxidative properties of nanocapsules. After illumination, dopamine is converted to leucine on the nanocapsules. Hence, nanocapsules can be used as photocatalysts to improve the photosensitization activity and photostability of phthalocyanine.

Camptothecin can induce tumor apoptosis by inhibiting the activity of topoisomerase I [90]. However, camptothecin has some major limitations in therapeutic applications, such as poor water solubility and rapid lactone ring hydrolysis at physiological pH values, which leads to inactive carboxylate forms [91–92]. Guo et al. [93] obtained water-soluble spiral nanofibers by coupling hydrophilic arginine and camptothecin. Self-assembly behavior is achieved by intermolecular π–π stacking and hydrophilic–hydrophobic interactions. The conjugates are linked by ester bonds, which help to maintain the camptothecin ring stability. The assembly can effectively enhance blood circulation, tumor accumulation and cellular uptake. In addition, arginine-modified camptothecin can be combined with anionic cisplatin–polyglutamic acid through electrostatic interaction to construct a co-delivery system.

**Table 1:** Summary of amino acids/amino acid derivatives and their applications.

Amino acids	Derivative/co-assembly	Forms of assembly	Applications	Ref.

H-Phe-OH H-Leu-OH	Fmoc-Phe-OH; Fmoc-Leu-OH	hydrogel	antibacterial	[45]

H-Phe-OH	Fmoc-Phe-OH; oligo(thiophene ethynylene)-ᴅ-phenylalanine	hydrogel	antibacterial	[47]

—	Fmoc-Lys(Fmoc)-Asp	hydrogel	2D/3D cell scaffolding; conductive composite hydrogels	[43]

H-Phe-OH	Fmoc-Phe-OH; aztreonam, an antibiotic drug	hydrogel	antibacterial	[48]

H-Phe-OH	Fmoc-Phe-OH; salicylic acid, a model drug	hydrogel	antibacterial	[49]

H-Phe-OH	Fmoc-Phe-OH; camptothecin, an anticancer drug; functionalized halloysite nanotubes, carrier for the camptothecin	hydrogel	drug delivery; antitumor	[50]

H-Phe-OH	NI-Phe-OH	hydrogel	live cell imaging in 3D scaffolding materials	[53]

H-His-OH	NI-His-OH	nanoparticles	bioimaging of Fe^3+^ ions; a selective diagnostic probe for cancer cells	[54]

H-Cys-OH	H-Cys-OH/Cd^2+^	nanorods	light harvesting, hydrogen evolution	[58]

H-Cys-OH	H-Cys-OH/Zn^2+^; TPPS^a^ and alcohol dehydrogenase, as model guest molecules	microspheres	biomimetic photosystems; chloroplast mimic	[59]

H-Cys-OH	H-Cys-OH/Zn^2+^; glutamate dehydrogenase, a guest molecule	microspheres	pigment model	[60]

H-His-OH	(Z^b^-His-NHNH_2_)/Zn^2+^	metallo-nanozyme	catalytic hydrolyzation	[62]

H-His-OH, H-Pro-OH, H-Ala-OH, H-Leu-OH	Fmoc-His-OH/Ag^+^, Fmoc-Pro-OH/Ag^+^, Fmoc-Ala-OH/Ag^+^, Fmoc-Leu-OH/Ag^+^	hydrogel	antibacterial	[68]

H-Leu-OH	Fmoc-Leu-OH/Mn^2+^; Ce6, a photosensitive drug	nanoparticles	drug delivery; antitumor; MRI	[70]

H-His-OH	Fmoc-His-OH/Zn^2+^, (Z^b^-His-Phe)/Zn^2+^; Ce6, a photosensitive drug	nanoparticles	drug delivery; antitumor	[71]

H-His-OH	Fmoc-His-OH/Zn^2+^; curcumin, an anticancer drug	nanoparticles	drug delivery; antitumor	[75]

H-Lys-OH	Fmoc-Lys-OH; H-Phe-Phe-NH_2_ HCl; Ce6, a photosensitive drug	nanoparticles	drug delivery; antitumor	[78]

H-Trp-OH; H-Gly-OH	tryptophan-glycine; porphyrin; a photosensitive drug	nanoparticles transform into nanofibers	drug delivery; antitumor	[83]

H-Phe-OH	*N,N'*-(disulfanediylbis(ethane-2,1-diyl))di-ʟ-phenylalamide; genipin, as crosslinking agent; indocyanine green, a photosensitive drug	nanoparticles	drug delivery; antitumor	[85]

H-Lys-OH	Fmoc-Lys-OH; TPPS^a^, model molecules of light-harvesting porphyrins	microspheres	light-harvesting, Hydrogen evolution	[87]

H-Lys-OH	Fmoc-Lys-OH; 3,4-dihydroxyphenylalanine; Sn(IV)tetrakis(4-pyridyl)porphyrin; Co_3_O_4_ NPs	nanofibers	oxygen evolution, biomimetic photosynthesis	[88]

H-His-OH	Fmoc-His-OH; phthalocyanine tetrasulfonic acid, a phthalocyanine model	nanovesicles	photocatalyst	[89]

H-Arg-OH	H-Arg-OH; camptothecin, an anticancer drug	nanofibers	drug delivery; antitumor	[93]

^a^Tetrakis(4-sulfonatophenyl) porphine; ^b^*N*-benzyloxycarbonyl.

## Conclusion

The self-assembly of biomolecules is based on the noncovalent interaction and the bottom-up combination of ordered 3D structures. Nanotechnology is the driving force of self-assembly, and it has made great contributions to the field of biology and biomedical science. The nanostructure of amino acids can be a good substitute for therapeutic delivery due to its good biocompatibility, functionalization, and ease of design/synthesis. Self-assembled nanostructures have become smart tools in the biomedical field, as demonstrated by the ability of self-assembled amino acid molecules to exhibit stimulation responsiveness to the environment, which has exciting prospects for use in drug delivery. The advantages of low production costs, easy dispersion in aqueous media, mild and rapid synthetic setup and simple functionality facilitate their use as future candidates for various applications such as drug delivery, imaging, diagnosis, and photochemistry. The morphology and structure of self-assembled nanomaterials can be flexibly adjusted by transforming the type, proportion, and concentration of the building blocks. This newer area of research is therefore accelerating. The assembly forms of amino acids include nanocapsules, nanoparticles, nanofibers, nanorods, nanoparticles, and hydrogels (Table 1). However, it is still a challenge to make these assemblies the preferred materials for scientific research and application. The difficulties of controlling the size and composition of nanostructures, the assembling behavior and stability in aqueous solution, the loading/encapsulation ratio of drugs, and the toxicity of nanostructures to living organisms still need to be overcome by researchers. Moreover, little research has been done on the biocompatibility of these nanostructures. Therefore, as a new strategy, amino acid self-assembly needs further research to explore the biomimetic and biomedical applications of micro- and nanomaterials.
